# Parental Influence

**Published:** 1873-02

**Authors:** 


### HALL'S

### JOURNAL OF HEALTH.

VOL. XX. — FEBRUARY, 1873. — NO. II.

### PARENTAL INFLUENCE.

IN France, one person out of every thirteen hundred becomes subject to legal punishment. But of infants abandoned in the streets by their mothers, one out of every one hundred and fifty-eight reaches the State Prison. With this view, eminent men have advised that it would be better to let them all die. But this fact shows the importance of parental training. The parents mould the character of their children, not only after they are born, but before. It is the taint in the blood, the mental and moral conditions of the mother while the infant feeds on her milk. A case lately occurred where the mother became uncontrollably enraged at her husband. In half an hour she calmed down and put her infant to the breast; it fell into convulsions, and died.

Other cases are given, leading to the inference that if within an hour or two of any violent mental emotion the impregnating act follows, the offspring has that predominant trait through life. Nothing else so well accounts for the diversity of character among children of the same parents The idea merits thoughtful consideration, that a temporary condition of the mind, of a very decided character, impresses itself on the offspring. That condition of mind may not be common to either parent, may not exist once in a year, but its existence gives the tinge, the hue to temperament and constitution. Aaron Burr's father was a clergyman, the son of a clergyman, and of irreproachable character his mother the daughter of a clergyman, of mind and morals and social position, nowhere excelled, seldom equalled The youthful pair were brought up in all the innocence and purity of a model family of educated, elevated Christian principles. But impure thoughts come to all at times; so do doubt and infidelity to the Christian faith. These may have existed, at critical times during gestation in the mother, or previously, in the father; for the offspring was a compound of mental power and moral degradation, villainous, traitorous, unprincipled, and impure.

